# Geographical inequality in service utilization for primary aldosteronism screening: spatial epidemiological study in Southern Thailand

**DOI:** 10.1186/s12913-022-07788-8

**Published:** 2022-04-07

**Authors:** Onnicha Suntornlohanakul, Suporn Sakarin, Noppadol Kietsiriroje, Hutcha Sriplung

**Affiliations:** 1 Endocrinology and Metabolism Unit, Division of Internal Medicine, Faculty of Medicine, Prince of Songkla University, Hat Yai, Songkhla, 90110 Thailand; 2 Department of Epidemiology, Faculty of Medicine, Prince of Songkla University, 15 Karnjanavanich Road, Hat Yai, Songkhla, 90110 Thailand

**Keywords:** Geographical inequality, Primary Aldosteronism, Screening, Thailand, Spatial epidemiology

## Abstract

**Background:**

Primary aldosteronism (PA), the most common cause of secondary hypertension is considered as a “major public health issue” due to higher risk of cardiovascular complications compared to blood-pressure-match hypertension and increase in prevalence around the world. In Thailand, though PA screening is provided under the universal health coverage, the service can be offered only at some centers. Hence, the service availability affects an accessibility of health care in patients. Our study aimed to evaluate the service utilization in PA screening and diagnosis in terms of geographical inequality in health resources in Southern Thailand.

**Methods:**

Data of 688 patients who underwent PA screening from 2011 to 2017 were obtained from the electronic database of Songklanagarind Hospital, a super-tertiary center in this region. The patients’ residence in the province, district and subdistrict were transformed to a 6-digit numbers corresponding to the global one (GADM©). The areas with PA screening and diagnosis were visualized by disease mapping procedures. A general log linear model was used to identify the factors affecting patient’s service accessibility.

**Results:**

From the geographic distribution, patients living in or near the area of the super-tertiary center (Songkhla) had high probability of receiving PA screening. The analysis of factors contributing to PA screening by multivariate log-linear model demonstrated that the distance from the super-tertiary center was a predictive factor for screening while the presence of endocrinologists and cultural differences were not. The chance of patients living in Songkhla, living less than 200 km, and more than 200 km from Songkhla to receive PA screening was 100, 82, and 66%, respectively. The crude incidence rate of PA in Southern Thailand was 1.66/10^6^ person-years. The provinces located adjacent to the Andaman Sea had the highest incidences of PA (3.62-5.17 patients/10^6^ person-years).

**Conclusions:**

There is still geographical inequality and the strategy to decrease the barrier should be resolved. The policymaker should develop a transfer system of blood tests for PA investigation from the local hospital to reduce the burden such as transportation costs in patients who live far away from the super-tertiary hospital. In addition, PA screening should be implemented in hypertension care plan.

**Supplementary Information:**

The online version contains supplementary material available at 10.1186/s12913-022-07788-8.

## Background

Primary aldosteronism (PA) is a disorder that has an inappropriately high production of aldosterone with suppressed renin level. The prevalence of PA has been a growing concern around the world and it is the most common cause of secondary hypertension accounting for 5-20% of hypertensive patients [1, 2]. In Southern Thailand, the PA prevalence of 16.7% has been reported in patients screened for secondary hypertension [3]. The early screening and diagnosis of PA are mandatory as PA significantly increases morbidity and mortality from cardiovascular disease and, if properly treated, the risk can be mitigated in the long-term [4–6]. Unfortunately, there remain some limitations in detecting hypertensive patients with PA [7].

The diagnosis of PA comprises three main steps: (i) screening, (ii) confirmation, and (iii) subtype classification. At the screening step, biochemical testing of plasma aldosterone concentration (PAC) and plasma renin activity (PRA) is needed. The patients with high PAC, suppressed PRA, and high plasma aldosterone-to-renin ratio (ARR) are considered as positive and the confirmatory tests are further conducted. If the diagnosis of PA is confirmed, adrenal venous sampling (AVS) and computer topography (CT) will be performed to differentiate between unilateral and bilateral aldosterone secretion. Finally, the patients with unilateral aldosterone secretion then receive adrenalectomy while those with bilateral aldosterone secretion receive mineralocorticoid receptor antagonists.

Geographic inequality of health services refers to the existence of geographic differences in health supplies and the utilization of health services whereby accessibility of healthcare is determined. In Thailand, inequality in the investigation of PA is demonstrated by the fact that biochemical testing, including PAC and PRA, is mainly available at a few tertiary centers [8]. On top of that, the accessibility of healthcare is subjected to several hindrances including financial constraints, organizational and personal barriers (such as attitudes, beliefs, and past experiences with healthcare services) [9]. These hindrances tend to have more effect to PA which is the complicated disease and need multiple hospital visits.

Thailand is a high-middle income country in Southeast Asia with 4 regions: Central, Northern, Southern and Northeastern, and over 70 million people. Southern Thailand, comprising 14 provinces, is separated into two coasts: East Coast (Gulf of Thailand) and West Coast (Andaman) and there are several mountain chains running through the Malaysian border. Hence, Southern Thailand’s culture is heavily influenced by its bordering country, Malaysia; consequently, the south is home to most of Thailand’s Muslims and Islamic influence.

According to Thailand’s public health insurance, there are two main stakeholders: public purchasers and service providers. The public purchasers are further divided into three groups with their respective health insurance schemes. Firstly, the Civil Servant Medical Benefit Scheme (CSMBS) managed by the Comptroller General Department (CGD) of the Ministry of Finance covers government employees. Secondly, the Social Health Insurance (SHI) managed by the Social Security Office (SSO) covers private workers of the Labour Ministry. Lastly, the Universal Coverage Scheme (UCS) covers the remaining population managed by the National Health Security Office (NHSO). Currently, these public health insurances cover the entire Thai population [10]. Most service providers in Thailand are public hospitals under the authority of the Ministry of Public Health (MOPH). The accessibility of healthcare mostly depends on public hospitals in the 13 health service regions of Thailand and 14 provinces of Southern Thailand are in the 11th and 12th health service regions.

**Fig. 1:**
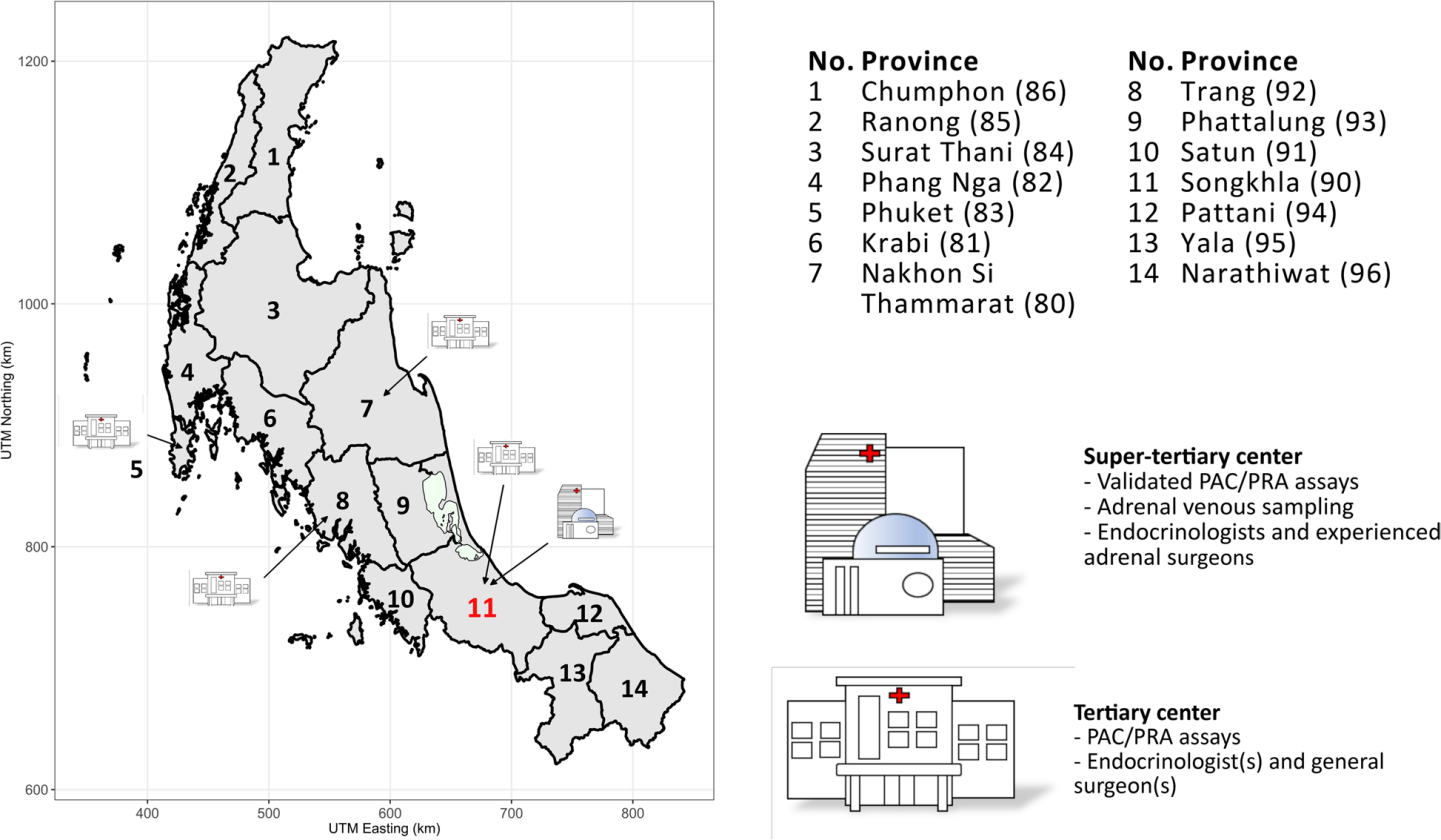
Provinces and the locations of tertiary and super-tertiary centers in Southern Thailand. Southern Thailand is in the 11th and 12th health service regions and comprises of 14 provinces (area code); Nakhon Si Thammarat (80), Krabi (81), Phang Nga (82), Phuket (83), Surat Thani (84), Ranong (85), Chumphon (86), Songkhla (90), Satun (91), Trang (92), Phattalung (93), Pattani (94), Yala (95), and Narathiwat (96). The map was generated by ggplot2 and other graphical packages in R software, using geographic data from DIVA-GIS (12)

The healthcare system of Thailand separates hospitals into multiple levels whereby patients with more complicated diseases are referred to a referral center (tertiary or super-tertiary center). In Southern Thailand, there are four public provincial hospitals acting as tertiary centers (Phuket, Nakhon Si Thammarat, Trang, and Hat Yai). Hat Yai which is the largest district in Songkhla also has a university hospital (Songklanagarind Hospital) which acts as a super-tertiary center (Fig. 1).

This study aimed to investigate the service utilization in detection and diagnosis of PA cases in Southern Thailand in terms of geographical inequality in health resources such as general physicians, endocrinologists, referral system and logistics. This study could be a pilot in the southern region, which can be extended to other regions in the country and elucidate the current situation and spatio-temporal trends of the disease in Thailand.

## Methods

### Data sources

This is a retrospective study using information collected from the electronic database - Prince of Songkla University Health Information System (PSU-HIS). All patients with the diagnosis of PA are eventually referred to Songklanagarind Hospital to undergo AVS, which is the final step for subtype classification.

### Study setting

The patients who have been referred to Songklanagarind Hospital can be representatives of PA screening and diagnosis in Southern Thailand.

### Inclusion and exclusion criteria

All patients who underwent calculation of the ARR as a screening tool for secondary hypertension from January 2011 to December 2017, were reviewed. Eligible cases were hypertensive patients (systolic blood pressure (SBP) > 140 mmHg and diastolic blood pressure (DBP) > 90 mmHg), which had measurements of both PAC and PRA. We excluded patients who underwent PAC or PRA measurement due to indications other than investigating the causes of secondary hypertension, aged less than 15 years, was a known case of PA, resided outside Southern Thailand, were on spironolactone during the tests, and had no confirmatory test.

### Sampling and sample size

As PA is rare disease, all patients who eligible to our study were included.

### Variables

#### Demographic variables

Baseline characteristics of the patients including age, sex, body mass index, blood pressure, etc. were extracted from PSU-HIS.

#### Patients’ residence

The patients’ residence was reviewed from their identification cards or the address recorded in the hospital system. The province, district and subdistrict of each patient were transformed to 6 digits of administrative area code corresponding with the global one (GADM©). The first, second and third two-digit codes represent province, district, and subdistrict, respectively.

#### Primary aldosteronism diagnosis

Demographic data, diagnostic tests (screening and confirmatory test for PA), AVS result and imaging study were reviewed independently by two physicians. Any discrepancies were solved by a consensus between the two physicians.

The diagnostic criteria of PA in our institute during the study period were (i) a PAC value > 15 ng/dl with a suppressed PRA value < 1.0 ng/ml/hr., and an ARR value > 30; and (ii) a positive confirmatory test result measured by either the Saline Infusion Test (SIT) or Fludrocortisone Suppression Test (FST), or alternatively the ARR > 100 [1, 11]. Details of PAC/PRA assays, confirmatory tests, and subtype classification, in our institute were given in Supplementary 1.

#### Predictor variables

The percentage of Muslim population was extracted from Thailand demographic data base. The number of endocrinologists in Southern Thailand was requested from Thai endocrine society. The distance between patients’ residence and Songklangarind hospital was calculated by Google map.

### Statistical analysis

Descriptive data were presented in mean ± SD, median (IQR), or number (%). The screening rates by district per year for PA were estimated by the total cases sent for screening throughout the 7-year period divided by the population of both sexes in the district in 2015 and multiplied by 7 years. Log base 10 of the rate was modeled with dependent variables using general log linear model with gaussian family link, and a normal distribution of the residual was confirmed by goodness of fit test. With a log-linear model, we found the predictors for individuals being screened. We estimated the incidence rate of true PA diagnosis in each province with the same procedure as described for screening rate. We computed the standardized rate ratio (SRR) for screening in provinces compared to the median screening rate with the following formula:

$SRR= OPR\left/ EPR\right.$, and the 95% standard error of the SRR was computed as:

$$seSRR=\sqrt{OPR}\left/ EPR\right..$$

Where observed provincial rate (OPR) was the estimated incidence rate by province as a result of the log-linear model mentioned above, expected provincial rate (EPR) was the median of incidence rate of all provinces obtained by the model. The difference between one level against the reference for a given dependent variable was presented as an absolute rate ratio, not an odds ratio, since the expected prevalence rates were computed from the model.

In plotting the spatio-temporal map of PA screening and diagnosis, we obtained the shape files for geographic coordinates of administrative areas (AMDA) from sub-district, district, and provincial levels (levels 3 to 1) from DIVA-GIS [12]. Disease mapping procedures were used to visualize the areas of PA screening and diagnosis. We ran the statistical computation and spatio-temporal mapping with R 3.6.2 using basic stats and graphic packages, and a series of specialized GIS packages.

The coordinates used on the maps were that of universal transverse mercator (UTM) zone 47 N / WGS 84. The projection lies between 96°E and 102°E, northern hemisphere between the equator and 84°N, onshore and offshore. We produced a map showing the distance in kilometers northward and eastward from the 0°N, 96°E coordinate. We handled the plots mainly with ggplot2 and ggmap packages. Using geom_polygon, the latitudes and longitudes at the subdistrict level were filled the results from the statistical models.

## Results

**Fig. 2:**
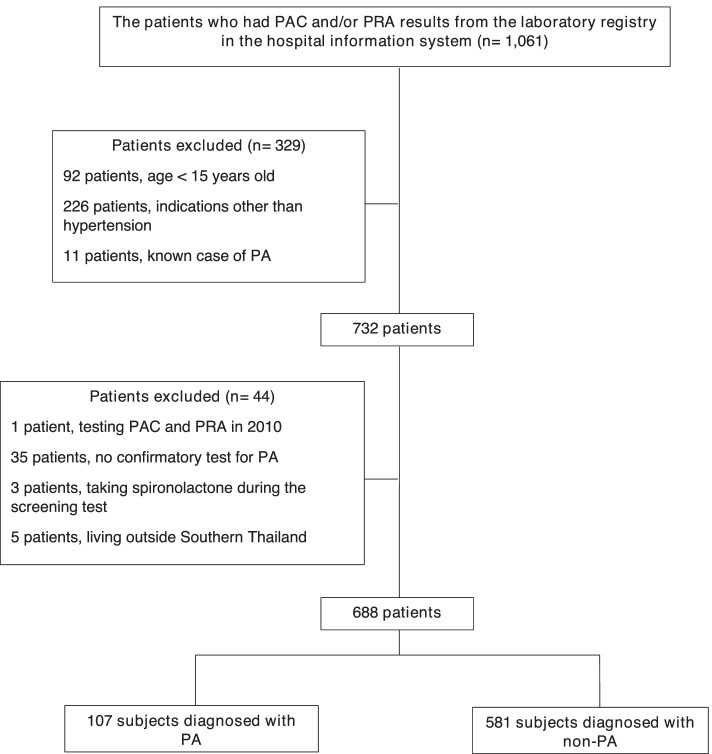
Flow of the study. (PAC, Plasma aldosterone concentration (ng/dL); PRA, Plasma renin activity (ng/mL/hr); PA, Primary aldosteronism)

Between January 2011 and December 2017, there were 1061 patients who underwent PAC and/or PRA testing in our hospital. After excluding patients, a total of 688 patients screened for PA were included in the analysis. The flow of study is shown in Fig. 2. Forty hundred and six patients (59%) were females with a median and IQR age of 63.3 (48.9, 78.5) years at the time when PAC/PRA were performed. The most common presentation was hypokalemia which is diagnosed in 298 patients (43.3%). Other baseline characteristics are described in Table 1.

**Table 1:** Baseline characteristics of the patients who were screened for and diagnosed with primary aldosteronism (PA)

	Median (IQR), no. (%)	
**Characteristics**	**Screened PA**	**Confirmed PA**
	**(*n*** **= 688)**	**(*n*** **= 107)**
Age, years	63.3 (48.9, 78.5)	63.7 (54.7, 75.3)
Sex, Female	406 (59.0%)	78 (72.9%)
Body mass index, kg/m^2^	25.3 (22.3, 29.0)	23.6 (21.3, 26.2)
Blood pressure, mmHg		
Systolic	149 (135, 163)	151.0 (139, 164)
Diastolic	89 (78, 98)	90.0 (80, 98)
Presentations		
Hypertension with hypokalemia	298 (43.3%)	98 (91.6%)
Hypertension in the young	175 (25.4%)	1 (0.9%)
Moderate to severe hypertension	97 (14.1%)	0 (0.0%)
Hypertension with adrenal incidentaloma	81 (11.8%)	4 (3.7%)
Resistant hypertension	23 (3.2%)	4 (3.7%)
Unknown cause	14 (2.0%)	0 (0.0%)
Serum potassium, mmol/l	3.8 (3.3, 4.1)	3.1 (2.7, 3.6)
Numbers of antihypertensive agents	1 (1, 2)	2 (2, 3)
Plasma aldosterone concentration, ng/dl	11.5 (5.2, 28.7)	56.6 (34.8, 91.8)
Plasma renin activity ratio, ng/ ml·h	1.2 (0.2, 4.6)	0.07 (0.01, 0.23)
Plasma aldosterone concentration to plasma renin activity ratio, ng/dl per ng/(ml·h)	7.1 (2.5, 34.8)	899.7 (219.4, 4155.0)

**Fig. 3:**
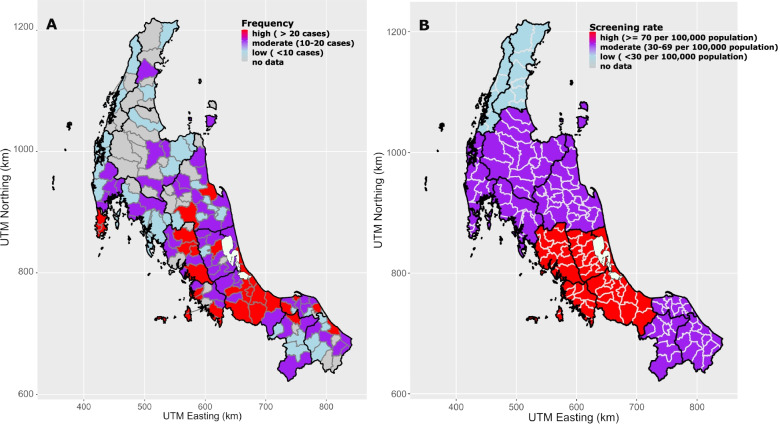
**A** Request of PA investigation by districts (**B**) The Screening rates of PA by provinces. (PA, Primary aldosteronism; UTM, Universal transverse mercator zone; Km, Kilometer), UTM zone 47 N, Indian 1975, EPSG:32647. The map was generated by ggplot2 and other graphical packages in R software, using geographic data from DIVA-GIS (12)

The proportion of patients who were screened for PA was highest in Songkhla (44.4%) and lowest in Ranong (0.3%), as shown in Table 2 and Fig. 3A (see also Fig. 1). Most patients who were screened lived in the major district of each province as shown in Fig. 3A and Supplementary 2.

**Table 2:** Numbers of screened and diagnosed, crude incidence rate of primary aldosteronism patients by province

Province	Screened PA	Diagnosed PA	Diagnosed PA among screened patients in each province	Crude incidence
(area code*)	**no. (%)**	**no. (%)**	**no. (%)**	**(per 1,000,000)**
				**(95%CI)**
Songkhla (90)	306 (44.4)	20 (18.7)	6.50%	2.03 (1.14-2.91)
Pattani (94)	57 (8.3)	13 (12.1)	22.80%	2.68 (1.22-4.13)
Nakhon Si Thammarat (80)	56 (8.1)	11 (10.3)	19.60%	1.01 (0.41-1.61)
Trang (92)	48 (7.0)	6 (5.6)	12.50%	1.34 (0.27-2.41)
Phatthalung (93)	46 (6.7)	10 (9.3)	21.70%	2.73 (1.04-4.43)
Satun (91)	45 (6.5)	8 (7.5)	17.80%	3.62 (1.11-6.12)
Phuket (83)	27 (3.9)	14 (13.1)	51.90%	5.17 (2.46-7.88)
Narathiwat (96)	27 (3.9)	6 (5.6)	22.20%	1.09 (0.22-1.97)
Yala (95)	25 (3.6)	4 (3.7)	16.00%	1.10 (0.02-2.18)
Surat Thani (84)	19 (2.8)	5 (4.7)	26.30%	0.68 (0.08-1.28)
Phang Nga (82)	14 (2.2)	7 (6.5)	50.00%	3.79 (0.98-6.59)
Krabi (81)	12 (1.7)	3 (2.8)	25.00%	0.93 (0-1.98)
Chumphon (86)	4 (0.6)	0 (0)	0.00%	0
Ranong (85)	2 (0.3)	0 (0)	0.00%	0
**Total**	688 (100)	107 (100)		1.66 (1.33-1.96)

area code; the two-digit codes represent each province, *PA* primary aldosteronism

**Fig. 4:**
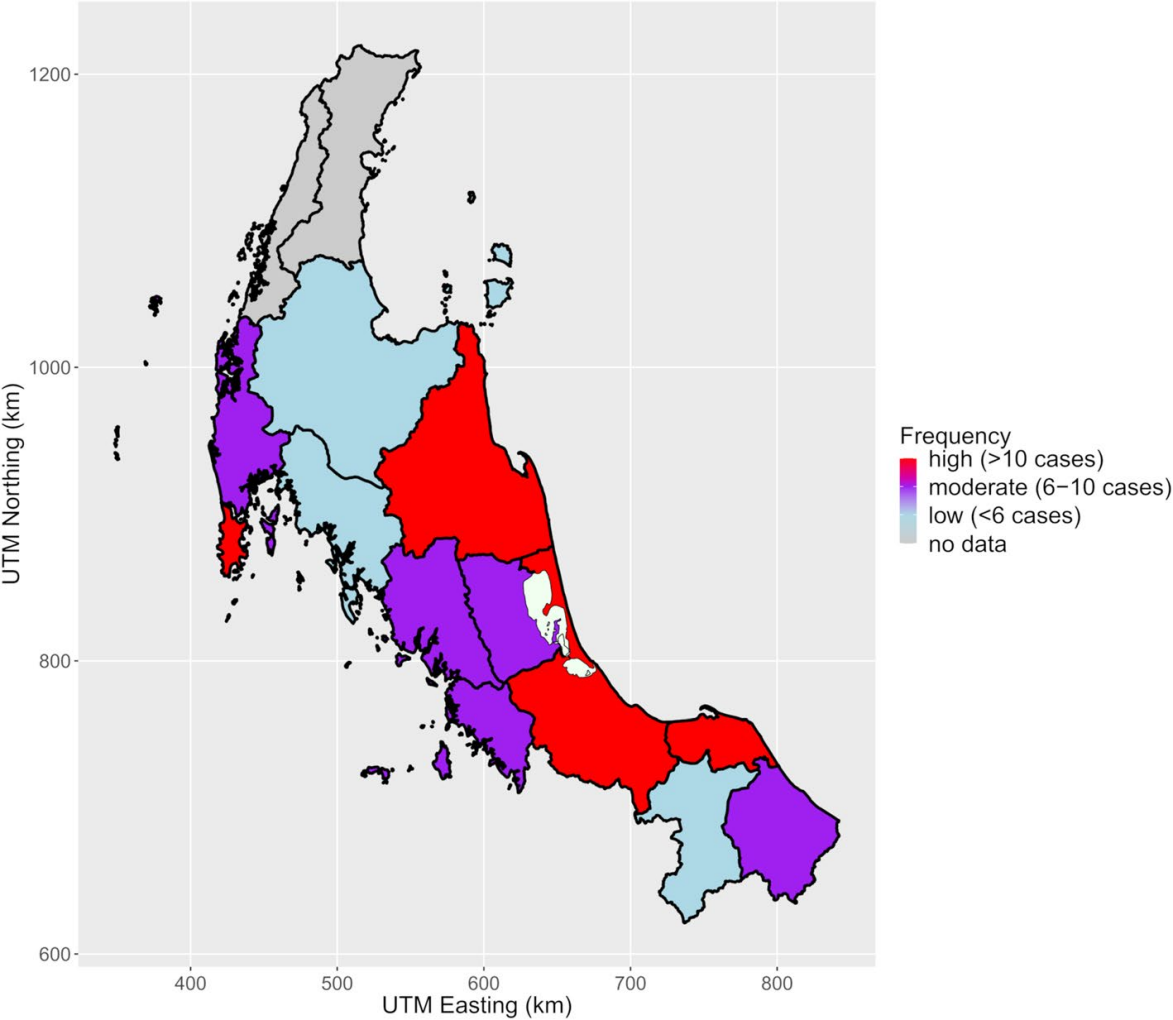
Numbers of patients who were diagnosed with primary aldosteronism by provinces. (UTM, Universal transverse mercator zone; Km, Kilometer), UTM zone 47 N, Indian 1975, EPSG:32647. The map was generated by ggplot2 and other graphical packages in R software, using geographic data from DIVA-GIS (12)

**Fig. 5:**
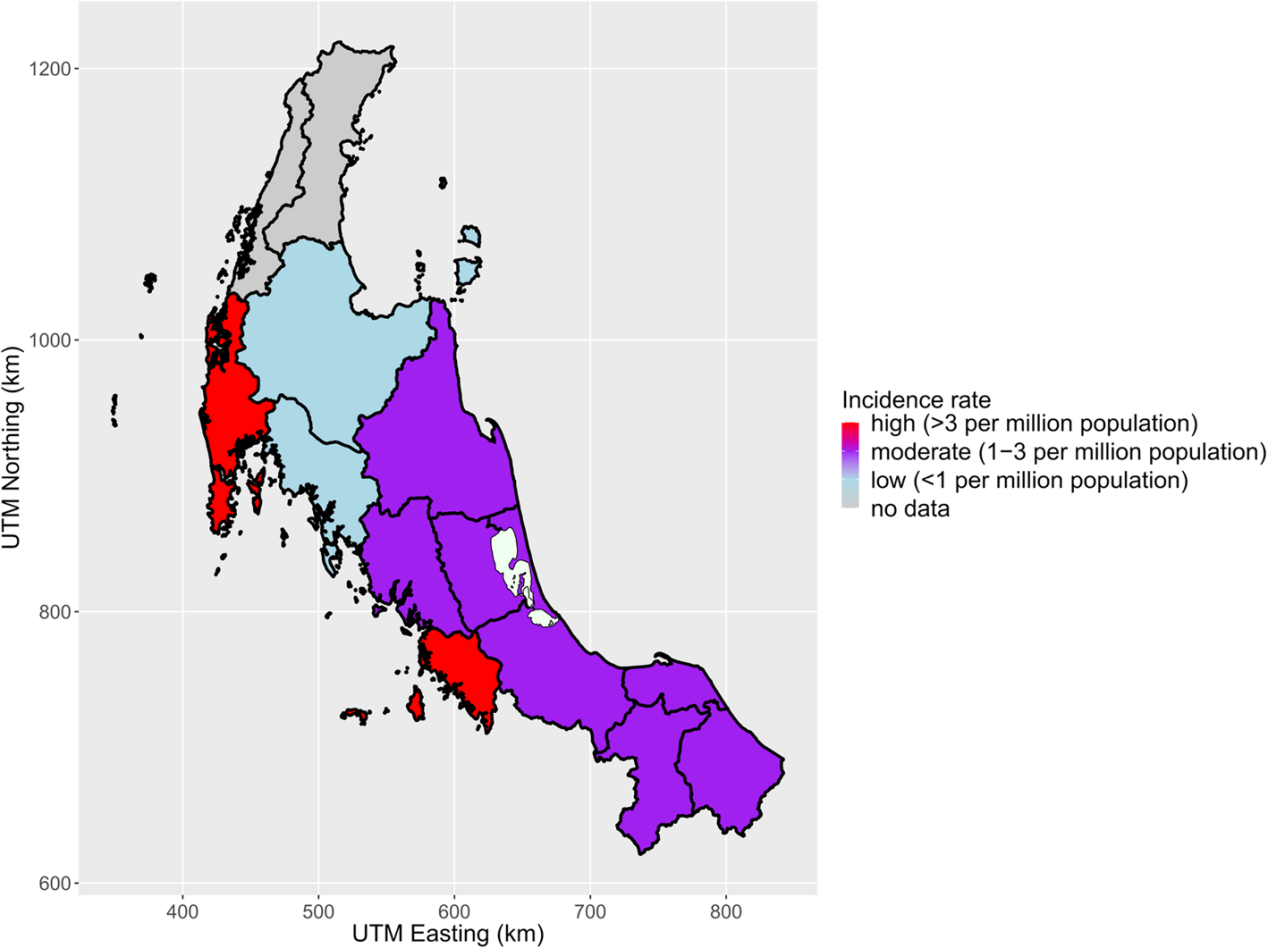
Incidence (per million population) of primary aldosteronism by province. (UTM, Universal transverse mercator zone; Km, Kilometer), UTM zone 47 N, Indian 1975, EPSG:32647. The map was generated by ggplot2 and other graphical packages in R software, using geographic data from DIVA-GIS (12)

A total of 107 (15.55%) patients were diagnosed with PA. The largest numbers of PA diagnosed cases were residents of Songkhla (20 cases) followed by Phuket (14 cases) and Pattani (13 cases) as displayed in Table 2 and Fig. 4. The incidences of the disease were highest in Phuket (5.17 (95%CI: 2.46-7.88) patients/10^6^ population) followed by Phang Nga (3.79 (95%CI: 0.98-6.59) patients/10^6^ population) and Satun (3.62 (95%CI: 1.11-6.12) patients/10^6^ population) (Table 2 and Fig. 5).

We performed a general log linear model where log10 of PA case detection was predicted by the presence of endocrinologist, distance from Songkhla, and the percentage of Muslim population while type I and type II errors were set at 0.05 and 0.20, respectively. The results showed that the distance from Songkhla and the presence of a Muslim population of more than 20% in the province were predictive factors for PA screening, while the presence of endocrinologists was not (Table 3). However, after the further analysis using multivariate log-linear model, the distance from Songkhla was the only predictive factor for PA screening. Supposing that living in Songkhla would have a rate of PA screening at 100%, living less than 200 km and more than 200 km away from Songkhla would have a rate of PA screening at 82% (95%CI:69-97%, *p*-value 0.03) and 66% (95%CI:55-78%, *p*-value < 0.001), respectively (Tables 3, 4 and Fig. 3B).

**Table 3:** Models analyzing factors contributing to the rates of PA case detection

Geographic factors	Univariate log-linear model	***P***-value	Multivariate log-linear model	***P***-value
	coefficient^**a**^		coefficient^**a**^	
	(95%CI)		(95%CI)	
**Areas with endocrinologists**	1			
Presence vs absence	1.02 (0.99-1.04)	0.084	–	
**Lives within Songkhla**^b^	1		1	
< 200 km away	0.80 (0.67-0.95)	0.012	0.82 (0.69-0.97)	0.03
≥200 km away	0.64 (0.53-0.76)	< 0.001	0.66 (0.55-0.78)	< 0.001
**Muslim**	1			
> = 20% vs < 20%	1.17 (1.02-1.33)	0.023	1.12 (0.99 -1.27)	0.08

^a^coefficient and 95%CI are derived from the standardized rate ratio (SRR) mentioned in the method section

^b^Songkhla is a province where the super-tertiary center with the largest number of endocrinologists are allocated.

Log-linear model = log_10_(rate) ~ x_i_, where x_i_ are the variables of interest

**Table 4:** Predicted screening rate of primary aldosteronism in 2015 in each province

Province	Predicted screening rate
**(area code**^**a**^**)**	**per 10**^**5**^**population**^**b**^
	**(95%CI)**
Songkhla (90)	114.8 (56.2-229.1)
Satun (91)	107.2 (45.7-257.0)
Trang (92)	81.3 (36.3-182.0)
Phatthalung (93)	77.6 (37.2-166.0)
Pattani (94)	57.5 (26.9-123.0)
Phang Nga (82)	49.0 (21.9-107.2)
Phuket (83)	47.9 (16.6-134.9)
Krabi (81)	38.9 (17.0-89.1)
Narathiwat (96)	38 (17.8-81.3)
Yala (95)	35.5 (15.5-81.3)
Nakhon Si Thammarat (80)	30.9 (23.4-40.7)
Surat Thani (84)	30.2 (14.1-63.1)
Chumphon (86)	18.6 (7.1-49.0)
Ranong (85)	10.2 (2.1-33.9)

^a^area code; the two-digit codes represent each province.

^b^Predicted screening rates are the predicted values of the log-linear model to the power of 10,

Log-linear model = log_10_(rate) ~ x_i_, where x_i_ represents province.

## Discussion

This study demonstrates that the distance from the super-tertiary center is the most important factor for the patients’ chances of receiving PA screening. Whereas the presence of specialist (endocrinologist) and the cultural differences are not. Meanwhile, the provinces located on the west coast adjacent to the Andaman Sea (Phuket, Phang Nga, and Satun) have the highest incidence of PA.

The effect of distance or geographical barrier to healthcare services is supported by previous studies conducted in both developing and developed countries [13–16]. Costs and difficulty in transportation might explain this finding. Although public health insurance covers most of the hospital expenses such as medical services and medicines, which are explicit costs, there remain the implicit costs that still put a burden on the patients. In general, the patients have to pay for transportation costs, and additional costs for accommodation in cases requiring an overnight stay. The government and other insurance schemes do not subsidize all these invisible costs, and they tend to be higher for the patients living far away from the healthcare center. That is, the farther they live, the more they pay. Since tertiary or super-tertiary centers are usually located in an urban area, with a middle- to high-income population, the most affected patients are the poor from rural areas. Likewise, public transportation is not available for all routes and times. Under these circumstances, some patients might refuse a referral to the healthcare center as it is accompanied by a heavy burden.

The spatio-temporal technique clearly visualizes the disease distribution in space and time in communicating disease, non-communicating disease, and climate effects on health [17, 18]. The finding from the spatio-temporal study could encourage the policymaker or government to allocate resources and budgets in appropriate area and time. Moreover, the spatio-temporal technique is a visualization tool illustrating the geographical inequality which should be figured out as it is the Sustainable Development Goal 10 (SDG 10; Reduced inequalities) of United Nations (UN) [19].

Geographic inequality as a component of SDG 10 mainly determined the access to health care. However, the solutions to socio-economic inequality can be grouped in to organizational and individual approach. The organizational approach has been implemented successfully in COVID-19 crisis. The approach includes 50-50 co-payment scheme and income subsidy both of which can be transformed to other group of affected patients [20]. The organization approach requires target population databases. The individual approach is to empower communities in Thailand to take care of underprivileged members. Thailand is among the leaders of primary health care scheme, and there will be a lot of evolution in our health care system which was developed during the COVID-19 crisis.

Nevertheless, low screening rates in Ranong, Chumphon, and Krabi could be attributable to the presence of alternative healthcare centers in Bangkok, the capital city of Thailand. The patients from these three provinces can easier access Bangkok than Songkhla by road and air. The decision of patients to obtain diagnosis and treatment outside their health care region is another effect of the distance.

The absence of the association between PA screening and the availability of endocrinologist suggests two possibilities. The physicians may be aware of PA while taking care of hypertensive patients, and also the existing referral system is active. There is also no effect of religious and cultural beliefs on PA screening. The phenomenon suggests that the investigation process of PA does not involve sensitive religious and cultural issues as it does in cervical cancer screening in terms of sexual exposure and reproduction, which leads Muslims to be reluctant to seek health care [21, 22].

It is noticeable that the population living in provinces adjacent to the Andaman Sea has high incidence of PA, even though they have a lower chance of receiving PA screening than people living in Songkhla or other nearby areas. The contributing causes should be assessed from both external and internal factors. One possible external factor is that these three provinces are popular tourist destinations with excellent transportation facilities and have high per capita income. Therefore, the people living in this area might have a better opportunity to gain access to the super-tertiary center. It should be noted that Phuket has a large medical center that can readily perform both screening and confirmatory tests of PA investigations by endocrinologists. Hence, the patients in Phuket and Phang Nga with negative screening tests were unlikely to be referred to Songklanagarind Hospital and the estimated screening rates using the PAC/PRA results in our center might be underestimated. Likewise, there is a high probability of having PA in patients who were referred to our hospital, leading to the selection bias of cases. Nonetheless, this is not evident in Satun, a small province further south towards Songkhla on the border with Malaysia, where Songklanagarind Hospital is the nearest and biggest hospital accessible to the patients.

Regarding the internal factors, people living in this area include the islanders in the Andaman Sea, such as sea gypsies with different genetic transmission from most Thais. These groups of islanders tend to have a diverse society and culture, i.e., consanguinity. Regrettably, we did not collect these details, and additional data evaluating this population should be studied in the future.

The strength of our study is that it was a spatial epidemiological study representing Thailand’s health inequality despite the presence of various public health insurance covering the entire Thai population. The results of this study could encourage policymakers both public purchasers and service providers to develop a strategy, which decreases barriers to healthcare access. For example, there should be a transfer system of blood tests for PA investigation, including teleconsultation to interpret laboratory results. The goal of the change is to reduce the number of patients being referred to the super-tertiary hospital. Developing a predictive scoring system of PA is also another good case to be implemented [3]. However, the current scoring system needs further validation study before being applied to routine clinical practice. According to the current hypertension screening program in Thailand, there is still limitation in effective coverage in hypertension screening management [23]. Future development of the program should be proposed together with incorporating PA screening into the program of MOPH, especially patient with hypertension in the young to investigation the secondary causes.

### Limitations

There are some limitations to this study. Firstly, it represented only Southern Thailand and could not be applied to other areas with different nationality, geographic area, and the healthcare system. Secondly, the incidence of PA in some provinces, especially Ranong, Chumphon, and Krabi, might be underestimated as the patients had a probability to be referred to Bangkok. Thirdly, the information about PA subtype, genetic study, and details of socio-economic-cultural data were not collected as described above. Further research about these details and other factors such as compliance and adherence to the system should be conducted in the future. In perspective of the health care system, other diseases which need sophisticated protocol including screening, confirmation test for diagnosis should be assessed in terms of feasibility, compliance, maintenance, and cost utility.

## Conclusion

In conclusion, the spatiotemporal pattern of PA screening shows a high activity around the super-tertiary healthcare center of Songklanagarind Hospital, and the incidence of the disease is high on the Andaman coast. There is still inequality in accessibility to PA screening in Southern Thailand. Healthcare policy should aim to decrease the inequality barriers. The extension of study areas to other regions in the country to elucidate the national trends of the disease is possible as the primary data structure in the healthcare system in Thailand is standardized, and the statistical processes have been validated in this study.

## Supplementary Information

**Additional file 1.**

**Additional file 2.**

## Data Availability

The datasets used and/or analysed during the current study are available from the corresponding author on reasonable request.

## Abbreviations

PA: Primary aldosteronism
PAC: Plasma aldosterone concentration
PRA: Plasma renin activity
ARR: Aldosterone-to-renin-ratio
AVS: Adrenal venous sampling
CT: Computer topography
CSMBS: Civil Servant Medical Benefit Scheme
CGD: Comptroller General Department
SHI: Social Health Insurance
SSO: Social Security Office
UCS: Universal Coverage Scheme
NHSO: National Health Security Office
MOPH: Ministry of Public Health
SBP: Systolic blood pressure
DBP: Diastolic blood pressure
SIT: Saline infusion test
FST: Fludrocortisone suppression test
SRR: Standardized rate ratio
UTM: Universal transverse mercator
SDG: Sustainable development goal
UN: United nations
