# Supplementary material for: Geographical inequality in service utilization for primary aldosteronism screening: spatial epidemiological study in Southern Thailand
Source: BMC Health Serv Res. 2022 Apr 7;22:458. doi: 10.1186/s12913-022-07788-8 (PMC8988538; doi:10.1186/s12913-022-07788-8)
Supplement: Supplementary file 1 — Additional file 1. [file 12913_2022_7788_MOESM1_ESM.docx]

**Supplementary 1.** Details of confirmatory tests and aldosterone and renin assays.

**Confirmatory tests** The saline infusion test (SIT) was performed between 0800-1200 am after an overnight fast. Patients remained in upright position for 2 hours prior to the infusion and then, were placed in the supine position throughout the testing. Two litres of isotonic saline were infused constantly though a four-hour period. Blood samples for serum electrolytes, plasma aldosterone concentration (PAC) and plasma renin activity (PRA) were drawn before and at the end of the infusion (1).

Fludrocortisone suppression test (FST) was used as an alternative confirmation to the SIT test when the SIT was contraindicated or unavailable. Patients received 0.1 mg oral fludrocortisone every 6 hours for 4 days, along with KCl supplement to keep serum K+ close to 4.0 mmol/l, and NaCl tab supplement (100 mmol/day of Na+). Blood samples for upright PAC and PRC were drawn in the morning (0800-1000) after patients had remained upright for at least 2 hours (2).

**Plasma aldosterone concentration and plasma renin activity assays**

Plasma samples were collected into EDTA (Ethylene diamine tetraacetic acid) tubes at room temperature. For economic reasons, the assay was performed every 2 weeks, samples were therefore stored at -20 °C for 7-11 days before the assays were performed. PAC was measured by radioimmunoassay (ALDO-RIACT, Cisbio Bioassays, Codolet/France). The intra-assay coefficients of variation (CV) were 11.7-13.6% during 2011-2014 and were 6.76-8.56% during 2015-2016. PRA was measured by radioimmunoassay (REN-CT2, Cisbio Bioassays, Codolet/France). The intra-assay CV were 10.3-15.5% during 2011-2014 and were 9.67-9.74% during 2015-2016.

**References**

1. Kem DC, Weinberger MH, Mayes DM, Nugent CA. Saline suppression of plasma aldosterone in hypertension. Arch Intern Med. 1971 Sep;128(3):380–6.

2. Mulatero P, Milan A, Fallo F, Regolisti G, Pizzolo F, Fardella C, et al. Comparison of confirmatory tests for the diagnosis of primary aldosteronism. J Clin Endocrinol Metab. 2006 Jul;91(7):2618–23.
