# Supplementary material for: Geographical inequality in service utilization for primary aldosteronism screening: spatial epidemiological study in Southern Thailand
Source: BMC Health Serv Res. 2022 Apr 7;22:458. doi: 10.1186/s12913-022-07788-8 (PMC8988538; doi:10.1186/s12913-022-07788-8)
Supplement: Supplementary file 2 — Additional file 2. [file 12913_2022_7788_MOESM2_ESM.docx]

**Supplementary 2.** Frequency of patients who were screened for primary aldosteronism in each district.

| **District (area code†)** | **Frequency of patients, no. (%)** |
| --- | --- |
| **Nakhon Si Thammarat (80)** |  |
| Mueang* (8001) | 16 (2.3) |
| Thung Song (8009) | 6 (0.9) |
| Lanska (8003), Chawang(8004),  Cha-uat (8007), Ron Phibun (8013) | 4 (0.6) |
| **Krabi (81)** |  |
| Mueang* (8101) | 4 (0.6) |
| Khao Phanom (8102) | 3 (0.4) |
| Ko Lanta (8103), Khlong Thom (8104),  Ao Luek(8105), Lam Thap(8107), Nuea Kholng (8108) | 1 (0.1) |
| **Phang Nga (82)** |  |
| Thap Put (8207) | 4 (0.6) |
| Mueang* (8201), Kapong (8203), Takua Thung (8204) | 2 (0.3) |
| Ko Yao (8202), Takua Pa (8205)  Khura Buri (8206), Thai Mueang (8208) | 1 (0.1) |
| **Phuket (83)** |  |
| Mueang* (8301) | 13 (1.9) |
| Thalang (8303) | 8 (1.2) |
| Kathu (8302) | 6 (0.9) |
| **Surat Thani (84)** |  |
| Mueang* (8401) | 5 (0.7) |
| Wiang Sa (8415), Phunphin (8417) | 3 (0.4) |
| Ko Samui (8404), Phrasaeng (8416) | 2 (0.3) |
| **Ranong (85)** |  |
| Mueang* (8501), Kra Buri (8504) | 1 (0.1) |
| **Chumphon (86)** |  |
| Sawi (8607) | 2 (0.3) |
| Pha Tio (8603), Lang Suan (8604) | 1 (0.1) |
| **Songkhla (90)** |  |
| Hat Yai** (9011) | 148 (21.5) |
| Mueang* (9001) | 52 (7.6) |
| Sadao (9010) | 27 (3.9) |
| **Satun (91)** |  |
| Mueang* (9101) | 22 (3.2) |
| La-ngu (9105) | 13 (1.9) |
| Khuan Don (9102), Thung Wa (9106) | 4 (0.6) |
| **Trang (92)** |  |
| Mueang* (9201) | 17 (2.5) |
| Huai Yot (9206) | 9 (1.3) |
| Na Yong (9208), Palian (9204) | 7 (1) |
| **Phattalung (93)** |  |
| Mueang* (9301) | 18 (2.6) |
| Pak Phayun (9306) | 5 (0.7) |
| Kong Ra (9302), Khuan Khanun (9305),  Pa Phayom (9310) | 4 (0.6) |
| **Pattani (94)** |  |
| Mueang* (9401) | 18 (2.6) |
| Khok Pho (9402) | 11 (1.6) |
| Sai Buri (9407) | 9 (1.3) |
| **Yala (95)** |  |
| Mueang* (9501) | 16 (2.3) |
| Betong (9502), Yaha (9505), Raman (9506) | 2 (0.3) |
| Bannang Sata (9503), Than To(9504), Kabang (9507) | 1 (0.1) |
| **Narathiwat (96)** |  |
| Mueang* (9601) | 9 (1.3) |
| Su-ngai Kolok (9610) | 5 (0.7) |
| Rueso (9606) | 3 (0.4) |

**†** Area code; The first and second two-digit codes represent province and district, respectively.

* Mueang is a name of major district in each province. The major district is a city where the city hall is located.

** Hat Yai is a district where the Songklanagarind Hospital (super-tertiary center) is located. In Songkhla, Hat Yai is bigger than Mueang.
